# Wavy changes in the whiskers of domestic cats are correlated with feline leukemia virus infection

**DOI:** 10.1186/s12917-023-03610-7

**Published:** 2023-03-04

**Authors:** Masataka Morishita, Yuji Sunden, Misaki Horiguchi, Hirosei Sakoya, Mana Yokogawa, Hiroyuki Ino, Satoshi Une, Mutsumi Kawata, Taisei Hosoido, Takehito Morita

**Affiliations:** 1 Niihama Animal Hospital, 2-1-11 Wakamizu Niihama, Ehime, 792-0017 Japan; 2 Neovets VR Center, 3-8-15 Nakamichi, Higashinari, Osaka 537-0025 Japan; 3 Laboratory of Veterinary Pathology, Joint Department of Veterinary Medicine, Faculty of Agriculture, Tottori University, 4-101 Koyama-Minami, Tottori, 680-8553 Japan; 4 Sigenobu Animal Hospital, 1054-1 Ushibuchi Touon, Ehime, 791-0213 Japan

**Keywords:** Feline leukemia virus, FeLV, Pathology, Wavy whisker (WW)

## Abstract

**Background:**

Feline leukemia virus (FeLV) is a retrovirus with global impact on the health of domestic cats and is usually examined by serology. In our daily clinical practice, we noticed that cats infected with FeLV often possess wavy whiskers (sinus hairs on the face). To investigate the relationship between wavy whiskers (WW) and FeLV infection, the association between the presence or absence of wavy changes in whiskers and serological FeLV infection was examined in a total of 358 cats including 56 cats possessing WW, using the chi-square test. The results of blood tests from 223 cases were subjected to multivariate analysis (logistic analysis). Isolated whiskers were observed under light microscopy, and upper lip tissues (proboscis) were subjected to histopathological and immunohistochemical analyses.

**Results:**

The prevalence of WW was significantly correlated with FeLV antigen positivity in the blood. Of 56 cases with WW, 50 (89.3%) were serologically positive for FeLV. The significant association between WW and serological FeLV positivity was also confirmed by multivariate analysis. In WW, narrowing, degeneration, and tearing of the hair medulla were observed. Mild infiltration of mononuclear cells in the tissues, but no degeneration or necrosis, was found. By immunohistochemistry, FeLV antigens (p27, gp70 and p15E) were observed in various epithelial cells including the sinus hair follicular epithelium of the whisker.

**Conclusions:**

The data suggest that the wavy changes in whiskers, a unique and distinctive external sign on a cat’s face, were associated with FeLV infection.

**Supplementary Information:**

The online version contains supplementary material available at 10.1186/s12917-023-03610-7.

## Background

Feline leukemia virus (FeLV) is a member of the genus Gamma retrovirus in the family of Retroviridae [1] and is classified into three subgroups according to differences in envelope proteins [2]. FeLV-A, the major subgroup, is involved in viral transmission between individual kittens and adults. FeLV-B and C occur in FeLV-A-infected individuals; FeLV-B is caused by recombination in the *env* gene between FeLV-A and the endogenous FeLV genome, and FeLV-C is derived from mutations in the *env* gene of FeLV-A [3, 4].

FeLV is associated with various disease conditions including tumors [5]. FeLV enters the body through oral or intranasal infection with virus particles contained in the saliva of FeLV-infected cats and mainly infects lymphocytes in the blood. Approximately half of infected cats may spontaneously eradicate the virus through the production of virus-neutralizing antibodies. These regressor cats become resistant to further infection. If the host's immune response is inadequate, the virus persists in the body (focal infection). However, approximately half of the progressor cats may never develop a clinical disease since a phase of viral persistence and latency in the bone marrow or in other body sites may occur [6]. When progressive infection develops, cats develope various disease conditions such as immunosuppression, anemia, malignant lymphoma and leukemia [6–9].

The mainstream methods for detecting FeLV are IFA-, ELISA- and/or PCR-based methods using blood [8, 9], and rapid detection kits have become widely available in recent years. Although some antiviral or immunomodulating drugs (e.g., interferon) are effective for FeLV infection [10], it is not easy to treat FeLV-infected individuals who develop severe disease. Therefore, preventing the spread of FeLV infection is important; infected cats must be identified and isolated to prevent contact with noninfected cats, and all cats that have the possibility of harboring the virus even if they do not show any abnormality, as recommended by the American Association of Feline Practitioners (AAFP) and the European Advisory Board on Cat Diseases (ABCD), respectively [8, 9]. However, FeLV infection is difficult to control because cats are often kept in large numbers and vertical transmission can occur from infected mothers to their kittens.

In our daily medical practice, we noticed that cats serologically positive for FeLV often possess wavy whiskers (sinus hairs) on the face. However, to the best of our knowledge, gross changes in the whiskers of animals have never been reported to date. The purpose of this study was to clarify the association between WW and FeLV infection.

## Results

### Association between WW and FeLV

**Fig. 1:**
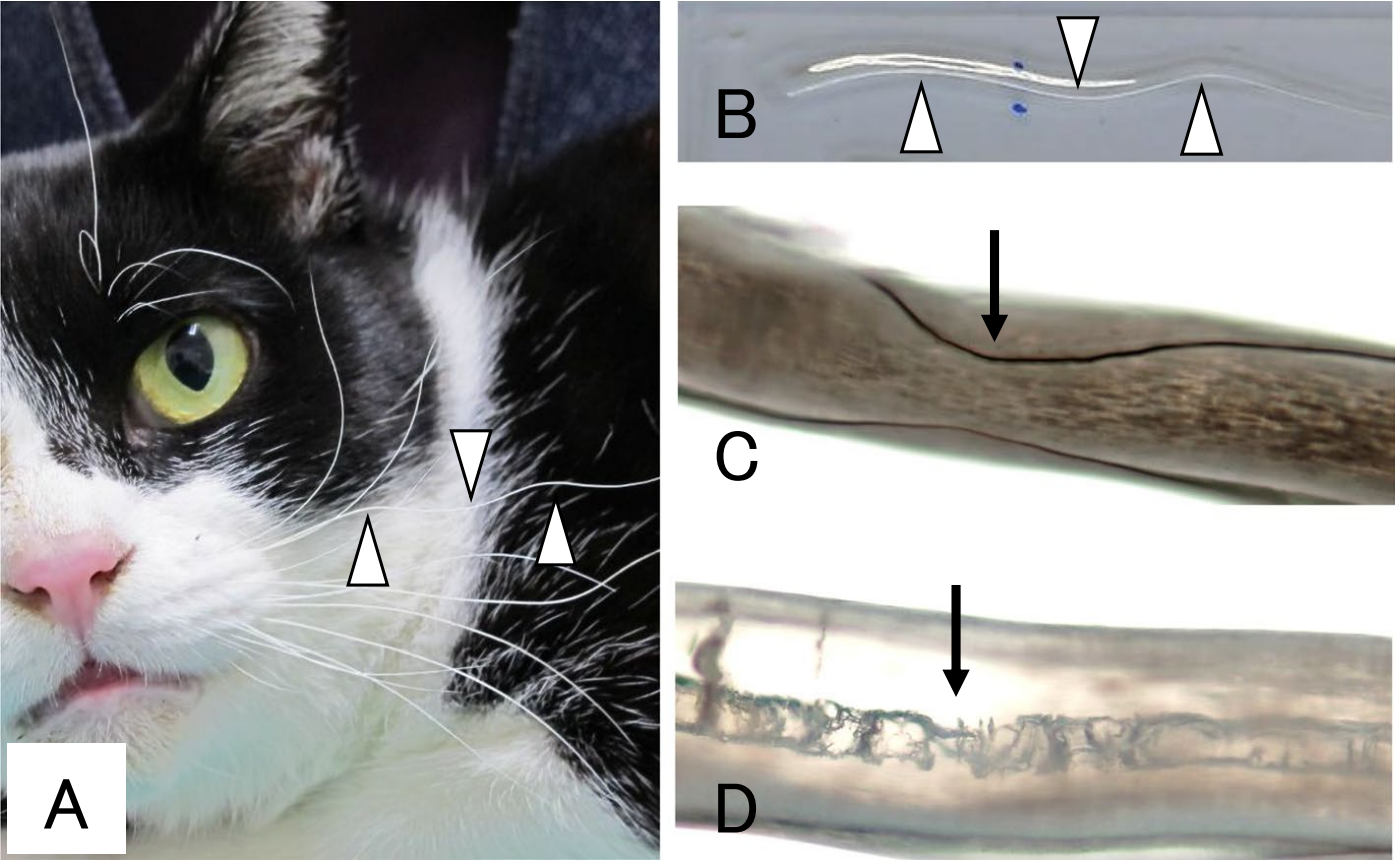
Gross and light microscopic appearance of wavy whiskers.** A** Curved and wavy whiskers on the upper lip of an FeLV-positive case. **B** An isolated whisker is fixed on a glass slide. Arrowheads show the bends in the hair. **C** The bent lesion of the hair shaft is shown under light microscopy as indicated by the arrow. **D** The ruptured medulla of the hair is also shown (arrow)

Wavy change was defined as more than two regions with bends in one whisker, and a cat possessing more than two whiskers with wavy changes was determined to be a WW-positive cat, with agreement among 3 clinicians. A typical appearance of WW is shown in Fig. 1A. There was a significant correlation between WW and serological FeLV positivity. Of the 56 patients with WW, 50 (89.3%) were positive for FeLV. Six cases had WW without serological detection of FeLV. These six cases were chronic renal failure (two), hemobartonellosis (one), trauma (one) and other diseases (two; not definitively diagnosed). A total of 302 patients had normal whiskers, and 289 (92.4%) patients were FeLV-negative by selorogy, 23 (7.6%) patients were FeLV-positive. Multivariate analysis showed a significant association between “FeLV infection (serological positive)” and “wavy whisker change” (*p* = 1.16E-11) (Table 1). The sensitivity, specificity, and positive predictive value were 68.4%, 97.9%, and 89.3%, respectively. Furthermore, the likelihood ratio between the serological FeLV test and WW positivity was 32.5 (> 10 has a significant contribution to the diagnosis [11]). Of a total of 50 cases, that were both serologically FeLV positive and WW positive, FeLV-related symptoms (anemia or lymphoma) were recorded in 17 cases (cases strongly suspected of FeLV involvement; 34%). Thus, a poor correlation between WW and symptoms was found, i.e., WW could be found even in nonsymptomatic or other diseased cats (33 cases; 66%). A statistically significant association with FeLV infection was also identified in low PCV (*p* = 7.63E-03).

**Table 1:** Summary of the multivariable logistic regression analysis, an objective variable as serological FeLV detection

	odds	95% confidence interval	*P* value	GVIF
WW	193	42.2–883	1.16E-11**	1.524867
Anorexia, lethargy	6.43	1.02–40.7	4.80E-02	1.461441
FIV positive	1.54	0.335–7.03	5.81E-01	1.228234
Castrared male (vs intact male)	0.197	0.037–1.04	5.62E-02	2.137209
Intact female (vs intact male)	0.212	0.0305–1.48	1.17E-01	
Spayed female (vs intact male)	0.602	0.141–2.58	4.94E-01	
BW	1.56	1.02–2.38	4.12E-02	1.493116
Age	0.923	0.787–1.08	3.23E-01	1.641185
PCV	0.919	0.864–0.978	7.63E-03*	1.405404
WBC	1	1	7.63E-01	1.157596
TP	0.916	0.464–1.81	8.00E-01	1.634495
ALT	1	0.996–1	8.29E-01	1.106538
Glu	0.998	0.986–1.01	7.86E-01	1.131883
BUN	0.99	0.964–1.02	4.64E-01	1.478275

WW: wavy whisker. GVIF, generalized variance inflation factor

*p<0.01

**p<0.001

Another important feline retroviral disease, feline immunodeficiency virus (FIV) infection [7, 8], was not associated with the prevalence of WW. In total, 36 serological FIV-positive cats were included in this study. There were 12 FeLV- and FIV- positive cats, of which 8 had WW and 4 had normal whiskers. FeLV-negative/FIV-positive cats (24 cases) had normal whiskers.

### Changes in WW and upper lip tissues

**Fig. 2:**
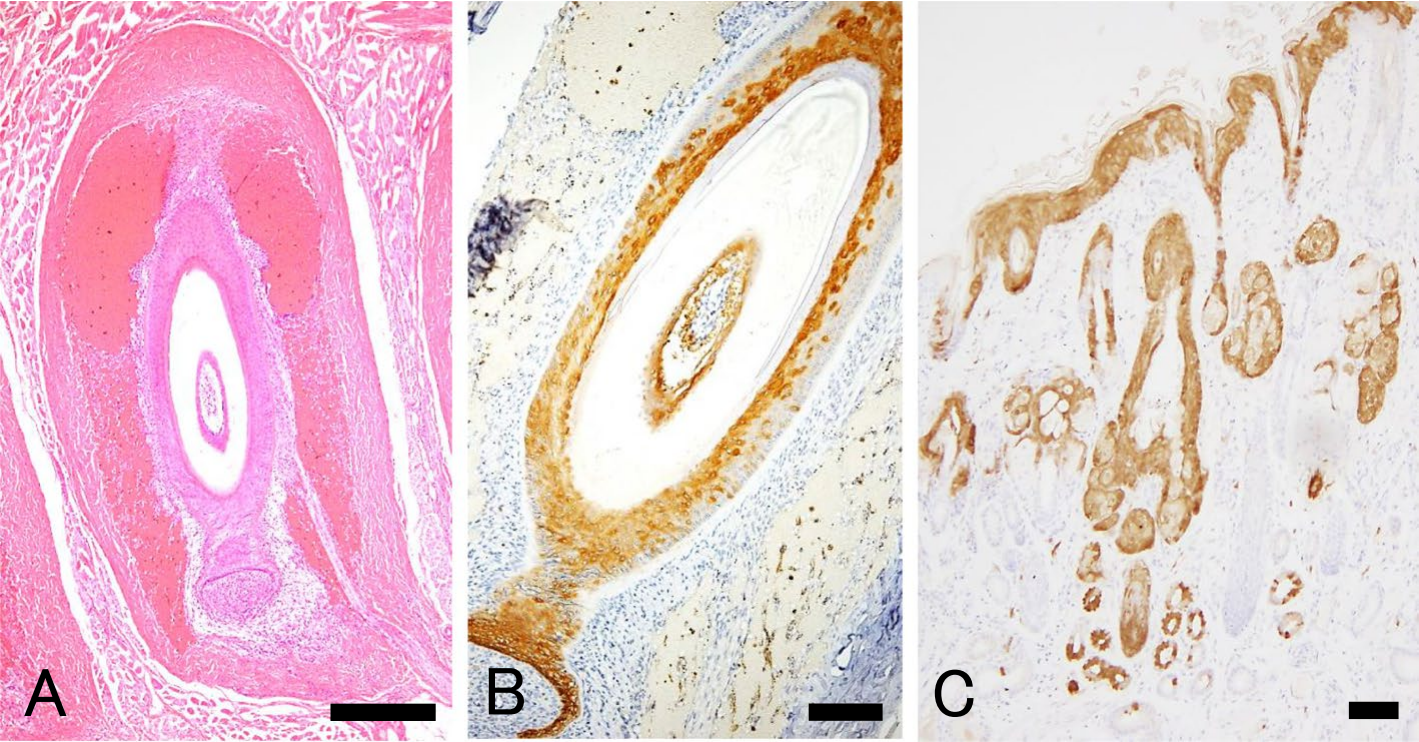
Histopathology of upper lip tissue and immunohistochemistry for FeLV p27 antigens.** A** No histopathological change was observed in the sinus hair follicle of the whisker. Large sinuses filled with erythrocytes are present around the follicle (normal structure). (HE stain). **B** The epithelial cells of follicular walls are strongly positive (brownish signals) for the p27 antigen of FeLV. The hair papilla, which consists of mesenchymal cells, is not stained (left lower angle). **C** The skin of the upper lip and the tissues around the whisker show positive p27 antigen staining, including the epidermis, sebaceous glands, and sweat glands. The nucleus was counterstained with hematoxylin (B and C). Bars = 500 µm (A and C), 250 µm (B)

In WW, narrowing, heterogeneity of diameter, and degeneration/tearing of the hair medulla were observed at the flexure points (Fig. 1B–D). No significant histopathological changes (e.g., degeneration and necrosis) were observed in the upper lip tissues (proboscis) of all cats (36 cases), except for a few mononuclear cell infiltrations in the dermis. Mild inflammation was observed in 11 of 13 serological FeLV-positive cats, and in 7 of 23 serological FeLV-negative or untested cats. Of 13 serological FeLV-positive cats, 6 cats had WW; however, no obvious lesion was observed in sinus hair follicles in all cats (Fig. 2A).

### FeLV antigen distribution in tissues

Immunohistochemistry (IHC) analysis detected antigen positivity in various epithelial cells of the proboscis, epidermis, mucosal epithelium, hair follicles, sweat glands, sebaceous glands, and the epithelium in sinus hair follicles of whiskers (Fig. 2B, C). Among the three FeLV antigens examined (p27, gp70, and p15E), p27 showed the highest detection rate. FeLV-p27 positive signals were often observed in epithelial cells of the tissues (epidermis, sweat gland, sebaceous gland, fair follicles of both skin hair and whiskers, and oral mucosa) in 12 cases (92%) from 13 serological FeLV-positive cases. Interestingly, five cases (38%) from 13 serological FeLV-positive cases had positive p27 signals in skeletal muscles. Of 12 FeLV-negative cases, only one case was positive for p27. The IHC data are summarized in Table 2.

**Table 2:** Summary of the immunohistochemistry (IHC) for each FeLV antigens

FeLV detection (serology)	WSH	FeLV antigen detection by IHC (upper: p27, middle: gp70, bottom: p15E)					
		Epi	Sw	Sb	Hf	Muc	Sm
Positive (*n* = 13)	6 (46)	12 (92)	11 (85)	12 (92)	12 (92)	10 (91)*	5 (38)
		9 (69)	11 (85)	8 (62)	8 (62)	10 (100)**	0 (0)
		5 (38)	3 (13)	0 (0)	1 (8)	0 (0)*	0 (0)
Negative (*n* = 12)	0 (0)	1 (8)	1 (8)	1 (8)	1 (8)	1 (8)	0 (0)
		0 (0)	0 (0)	0 (0)	1 (8)	1 (8)	0 (0)
		1 (8)	1 (8)	0 (0)	0 (0)	0 (0)	0 (0)
Not tested (*n* = 11)	0 (0)	0 (0)	0 (0)	0 (0)	0 (0)	0 (0)	0 (0)
		0 (0)	0 (0)	0 (0)	0 (0)	0 (0)	0 (0)
		0 (0)	0 (0)	0 (0)	0 (0)	0 (0)	0 (0)

The number of cases those had wavy whiskers (WW), FeLV antigens (p27, gp70 and p15E, respectively) in each region. The percentage is also listed in parenthesis. Epidermis (Epi), epithelium of sweat gland (Sw), sebaceous gland (Sb), hair follicles of both skin hair and sinus hair. Oral mucosa of upper lip (Muc) and muscles (Sm)

Eleven*

Ten**

Cases are tested

## Discussion

Although retroviral diseases in cats have diverse manifestations, most of them are associated with immunosuppression, anemia and lymphoma [5–7]. A low packed cell volume (PCV), detected in this study, is a marker of anemia, which is a typical symptom of FeLV-infected cats, suggesting that our caseload included enough samples and its reliability. This study is the first to show a significant association between WW and serological FeLV detection. The specificity and positive predictive value of WW to detect the presence of FeLV infection (the presence of FeLV antigen in the blood) were high, indicating that whisker changes may be a distinct external marker for FeLV-infected cats.

FeLV infection is commonly diagnosed using blood-based antigen detection [12]. However, because the symptoms of FeLV infection vary and the disease progresses slowly, it is difficult for owners to notice abnormalities in their cats. Therefore, the findings of this study, i.e., that the easily observable whiskers on faces can be a suggestive finding of infection without capturing or restraining the cat are important. However, the sensitivity was slightly low at 68.4%. This means that one-third of cases are false-negative results by WW-based judgment. In the present study, of a total of 73 serologically FeLV-positive cats, 50 had WW, and 23 had normal whiskers. Furthermore, the association between WW and the occurrence of FeLV-related symptoms is unclear in our cases. Therefore, WW judgment is not the most relevant screening test and serology is still preferable; however a combination of WW and serology could be an effective tool of FeLV infection for future applications.

FeLV is transmitted among individuals through body fluids (saliva, tears, and urine) [5–7, 13–15]. The virus-infected cells in the body are mainly blood cells (lymphocytes and monocytes) [16, 17], and the mechanisms of virus extravasation remain unclear. In this study, viral antigens were abundant in epithelial cells of the proboscis tissue, confirming the importance of these cells as a source of viral efflux into saliva [18]. In the present study, viral antigens were also detected in sebaceous and sweat glands, suggesting that FeLV may be excreted in exocrine fluids such as sebum and sweat. FeLV infection occurs in various stages with different outcomes [6, 19]. Viremic stages occur within a few weeks after the first virus exposure. After that, most of the virus localizes in the bone marrow, oropharyngeal lymphoid tissues and salivary gland. Serologically detectable viral proteins are released from localized areas without obvious progeny virion production, and some viral particles are also released in body fluids, including saliva [6]. Taken together with our results, although the detailed mechanism underlying the association of WW with FeLV remains unclear, WW might be formed over a period of several months after virus exposure, probably during the regressive, focal and progressive phase of infection [6], and viral replication in the epithelium of sinus follicles at the root of whiskers, without tissue injury, might be involved in the formation of WW.

The mononuclear cells infiltrating into the dermis consisted of a mixture of T lymphocytes, B lymphocytes, and macrophages (confirmed by immunohistochemistry) in 36 cases that were histopathologically analyzed in the present study. The significance of these infiltrating cells might be of little importance because FeLV-negative cases also had some inflammatory changes. Enlarged lymphoid organs and lymphocyte proliferation in blood were not observed, suggesting that no neoplastic changes occurred in these cats. Detailed and adequate examination of bone marrow in these cases was not performed, so the infection phase of these cats is unclear.

In this study, FeLV antigens were detected in the follicular epithelial cells of whiskers. Three FeLV envelope proteins, namely, p27, gp70 and p15E, were tested by IHC in this study. The viral core protein p27 is a common target for FeLV detection [20, 21], and among the three targets, p27 was the most frequently detected. Interestingly, p27 was also detected in skeletal muscle fibers in 35% (5 cases) of FeLV-positive cases in blood. There have been no previous reports of FeLV infection in skeletal muscle. However, it has been reported that FeLV-A enters cells using feline thiamine transport protein 1 (feTHTR1), which is distributed throughout the body, as a receptor and that the *feTHTR1* gene is also slightly expressed in skeletal muscles [22]. The significance of this finding remains unknown and is an issue for further study.

In a preliminary study with a few cases, virus-like particles were detected in the epithelium of sinus hair follicles of whiskers in FeLV-positive cats by transmission electron microscopy (Supplemental Information, Figure S1). This finding suggests that the infected viruses replicate in the epithelium and produce progeny virions. However, there was no morphological degeneration or necrosis of the cells comprising the follicular walls, suggesting that the wavy changes in whiskers caused by FeLV infection were not due to injury to the follicular wall. FeLV antigens were also observed in the skin hair follicular walls, although no abnormalities such as alopecia or wavy changes were observed in the whole body coat. This result suggests that the effect of FeLV infection in the hair shaft may be limited to the whiskers. The tendency of WW to bend at multiple sites suggests that wavy changes occurred as a result of the intermittent long-term effects of FeLV infection.

The reasons for the presence of WW in six cases of serologically FeLV-negative cats are unclear; however, IHC of these cats was not available in the present study, and the possibility of localized FeLV infection in the proboscis could not be completely ruled out. The one case with negative serology and positive IHC in tissue was also difficult to interpret; however, false-negative or technically erroneous serology and complex infection status could be possible.

A study focusing on the association between viral infection and sinus hair was reported for some cases of rabies [23]. The distribution of rabies virus in the sinus hair follicular wall showed that the target cells were Merkel cells, which are specialized sensory receptor cells in the sinus hair wall, and this localization could be related to the dense nerve distribution in these cells. Another study reported that murine leukemia virus can proliferate in the follicular wall of the skin [24]; however, the results were obtained by experimental subcutaneous virus inoculation of newborn mice, and the significance of this finding is unknown. To date, this is the first report showing the unique wavy changes in whiskers (sinus hair) associated with virus infection.

## Conclusion

To the best of the authors’ knowledge, this is the first study to focus on the wavy changes in whiskers in animals. The conclusion of this study was that wavy changes in whiskers, a unique and distinctive external sign on the face of domestic cats, were associated with FeLV infection and detection of viral p27 antigen in their blood and in sinus hair follicles. The mechanisms of wavy changes in whiskers and the detailed clinical phase of this phenotype are still unknown and further studies are required.

## Methods

### Case selection, serology and definition of wavy whiskers

To identify diseases present in cats with wavy whiskers (WW) on their faces, we recorded information from examinations of cats visiting Niihama Animal Hospital in Ehime Prefecture, Japan, during 2006–2013. A Significant wavy change was defined as more than two regions with bends in one whisker, and a cat possessing more than two whiskers with wavy changes was determined to be a WW-positive cat (Fig. 1A) with the agreement of 3 clinicians (MM, YM, and IH). To accumulate enough WW-positive cats, WW-negative cats were also randomly recorded. A total of 358 cats, including 56 with WW, were enrolled in the present study. Some of the available case information about clinical diagnosis, especially FeLV-related disease or not, was recorded.

### Clinical examination

Of 358 cats, 223 cases had recorded information available for the following examined items: signs of lethargy and anorexia, the presence of WW, body weight, sex, and age. Furthermore, packed cell volume (PCV), white blood cell (WBC) count, total protein (TP), glucose (Glu), alanine aminotransferase (ALT), and blood urea nitrogen (BUN) were detected by normal blood tests. Viral infection was determined using a rapid test kit (SNAP FIV/FeLV Combo Test, IDEXX Japan, Tokyo), which detects the viral antigen (FeLV p27, core protein of the virus) and anti-FIV-specific antibody.

### Statistical analysis

First, the associations between the presence or absence of WW and serological FeLV or FIV infection were determined by using the chi-square test [25]. Next, multivariate analysis (logistic analysis) [26] was performed to test the association between “serological FeLV detection” and the above clinical examination items. The sensitivity, specificity, positive predictive value and likelihood ratio were calculated between FeLV serology and WW positivity according to a previous report [11].

### Observation of WW

Thirteen serologically FeLV-positive cats were determined to have WW, and 9 FeLV-negative cats were determined to have normal whiskers. The samples were attached to a glass slide with clear tape, and the hair morphology was observed under a light microscope.

### Histopathology and immunohistochemistry

The upper lip tissues (proboscis) including the whisker follicles from 36 cases were formalin fixed, embedded in paraffin wax, and stained with hematoxylin and eosin (HE). Samples were collected humanly from cadavers with the owners’ consent and permission. IHC detection of FeLV antigens (p27, gp70 and p15E) was also performed. The primary antibodies used were the following mouse monoclonal antibodies: FeLV p27 (clone PF12J-10A, Abcam, Cambridge, England), gp70 (clone C11D8, Novus Biologicals, CO, USA), and p15E (clone PF6J-2A1, Novus Biologicals). Briefly, for antigen activation, each microsliced specimen was immersed in 0.01 M citrate buffer (pH 5.4) and microwaved at 98 °C for 15 min. Endogenous peroxidase activity was inhibited by performing the reaction in 3% hydrogen peroxide-containing methanol solution for 15 min at room temperature. To inhibit nonspecific reactions, slides were treated with Blocking One Histo reagent (Nacalai Tesque, Kyoto, Japan) and normal goat serum. All primary antibodies were used at a 300-fold dilution and reacted at 4 °C overnight. Phosphate buffer saline (PBS) was used as a negative control. After the primary antibody reaction, the slides were washed with PBS and incubated with peroxidase-conjugated goat anti-mouse antibody (Nichirei Bioscience, Tokyo) for 30 min at room temperature. Each antigen–antibody reaction was colorized with 3, 3'-diaminobenzidine and counterstained (nuclear staining) with hematoxylin.

## Supplementary Information

**Additional file 1: Fig. S1.** Transmission electron microscopy of the sinus hair follicular epithelium of an FeLV p27-positive case by serology and IHC. A Follicular epithelial cell with nucleus (N) and cytoplasm in the right upper area. B Magnified view of the area enclosed by the square in A showing the surface of the cell with a double-layered membrane. Some round virus-like particles are indicated by arrows. C Magnified view of the area enclosed by the square. Many round virus-like particles, approximately 80 nm, were observed within the cytoplasm. Bars = 1.0 µm (A), 200 nm (B) and 100 nm (C).

## Data Availability

The datasets used and analyzed during the current study are included in the article. More details of the information of the cases are not available for public access because of privacy concerns, but available from the corresponding author on reasonable request.

## Abbreviations

FeLV: Feline leukemia virus
WW: Wavy whiskers
IHC: Immunohistochemistry
PCV: Packed cell volume
FIV: Feline immunodeficiency virus
BW: Body weight
feTHTR1: Feline thiamine transport protein 1
WBC: White blood cell
TP: Total protein
Glu: Glucose
ALT: Alanine aminotransferase
BUN: Blood urea nitrogen
HE: Hematoxylin and eosin
PBS: Phosphate buffer saline
