# Supplementary figures and images for: Wavy changes in the whiskers of domestic cats are correlated with feline leukemia virus infection
Source: BMC Vet Res. 2023 Mar 4;19:58. doi: 10.1186/s12917-023-03610-7 (PMC9985215; doi:10.1186/s12917-023-03610-7)

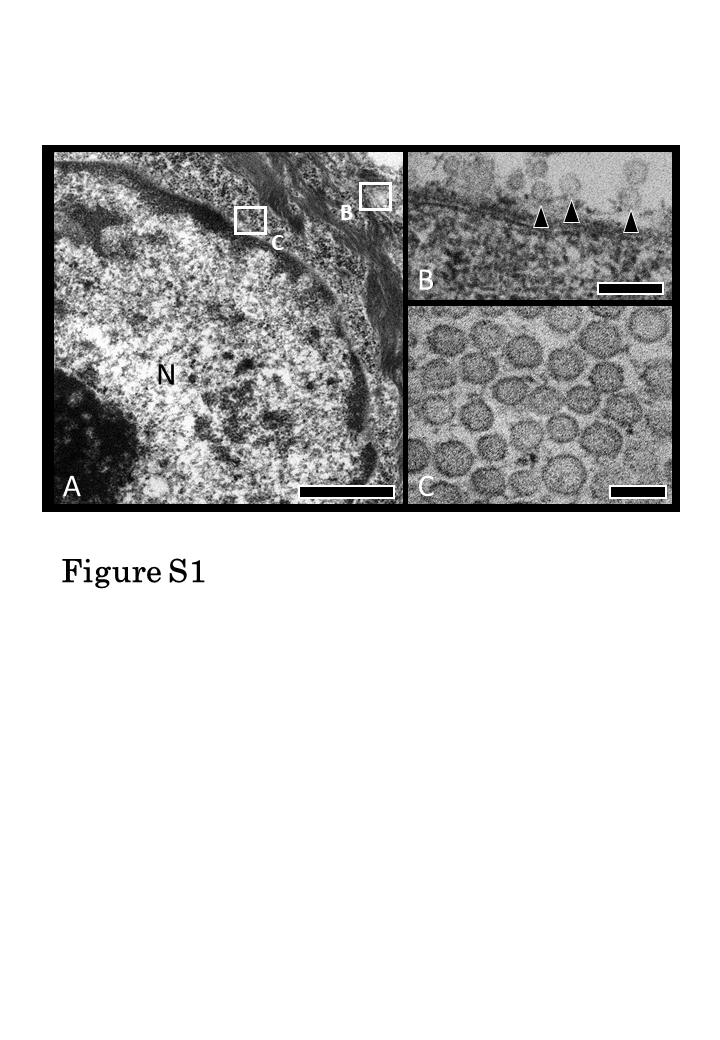

Supplement: Supplementary file 1 — Additional file 1: Fig. S1. Transmission electron microscopy of the sinus hair follicular epithelium of an FeLV p27-positive case by serology and IHC. A Follicular epithelial cell with nucleus (N) and cytoplasm in the right upper area. B Magnified view of the area enclosed by the square in A showing the surface of the cell with a double-layered membrane. Some round virus-like particles are indicated by arrows. C Magnified view of the area enclosed by the square. Many round virus-like particles, approximately 80 nm, were observed within the cytoplasm. Bars = 1.0 µm (A), 200 nm (B) and 100 nm (C). [file 12917_2023_3610_MOESM1_ESM.tif]
